# Smart Optical Catheters for Epidurals

**DOI:** 10.3390/s18072101

**Published:** 2018-06-30

**Authors:** Benito Carotenuto, Armando Ricciardi, Alberto Micco, Ezio Amorizzo, Marco Mercieri, Antonello Cutolo, Andrea Cusano

**Affiliations:** 1 Optoelectronics Group, Department of Engineering, University of Sannio, 82100 Benevento, Italy; benito.carotenuto@unisannio.it (B.C.); alberto.micco@unisannio.it (A.M.); cutolo@unisannio.it (A.C.); 2 Pain Medicine Unit, Sant’Andrea Hospital, “Sapienza” University, 00189 Rome, Italy; amorizzoezio@gmail.com (E.A.); marco.mercieri@uniroma1.it (M.M.)

**Keywords:** fiber optics sensors, fiber Bragg gratings, clinical applications, medical optics instrumentation

## Abstract

Placing the needle inside the epidural space for locoregional anesthesia is a challenging procedure, which even today is left to the expertise of the operator. Recently, we have demonstrated that the use of optically sensorized needles significantly improves the effectiveness of this procedure. Here, we propose an optimized configuration, where the optical fiber strain sensor is directly integrated inside the epidural catheter. The new design allows the solving of the biocompatibility issues and increases the versatility of the former configuration. Through an in vivo study carried out on a porcine model, we confirm the reliability of our approach, which also opens the way to catheter monitoring during insertion inside biological spaces.

## 1. Introduction

The epidural procedure consists of injecting the anesthetic in the outermost part of the spinal canal, a thin anatomic space called the epidural space (ES). Positioning the needle inside the ES is not trivial. Failure rates of up to 7% result from the fact that, currently, clinicians still adopt manual techniques whose success depends on their manuality and experience [1]. Errors in the ES identifications may involve serious inconvenience to patients and, indirectly, to the healthcare system [2]. There is an increasing interest in the scientific community in proposing alternative approaches and devices in order to enhance the success of the epidural procedures [3,4]. Over the last years, by taking advantage of the intrinsic properties of optical fibers in terms of their ease of integration inside medical needles, several optical approaches have been reported [5,6,7,8,9,10]. The fiber-based devices can be basically classified in two main categories, namely optical refractometers and optical coherence tomography (OCT) probes. The former relies on spectroscopy measurements [5,6,7] where each tissue is associated to a specific optical absorption coefficient at different wavelengths. The latter instead collect OCT images to discriminate among different tissues [8,9,10].

In this framework, we have recently proposed a guidance system based on the integration inside the needle lumen of a Hytrel-coated fiber Bragg grating (FBG) [11,12]. The FBG works as a strain sensor and measures the force exerted on the needle tip during the tissue penetration. The ES is identified when the optical sensor measures an abrupt force drop whose characteristics are unique, thus making the ES identification reliable and accurate. Our device has been successfully tested on a lumbar training phantom [11] and also on a porcine model [12]. However, the weak biocompatibility of Hytrel makes this device unsuitable for in vivo applications.

With the aim of overcoming this limitation, here we propose an optimized version of our device, where the FBG is integrated inside a conventional epidural catheter (EC) which, in its turn, is inserted inside the epidural needle. Interestingly, through in vivo tests, we demonstrate how the new device, being based on an optically sensorized catheter, is able to assist clinicians not only in the correct positioning of the needle but also of the EC inside the ES. This may seem a trivial aspect, but it is definitely not. In fact, in most of the epidurals, the drug injection is performed with the aid of the catheter; once the needle tip is in the right position, the EC is passed through the needle and it is placed into the ES. The needle is then taken out, and the EC is left in place, inside the patient’s back, to allow for intermittent or continuous infusion of drugs to relieve pain for prolonged periods. However, during the advancement, the catheter might deviate from its straight course and bend in such a way that the injected drug is not beneficial. This side-effect, known as catheter “kinking” or “coiling”, is very common in the clinical practice, and it represents the major cause of failed epidurals at more than 40% of cases [13]. We demonstrate that, if the EC bends during its advancement through the ES, so does the embedded optical fiber sensor, thus involving an intensity drop of the FBG back-reflected signal [14]. Overall, the proposed device has the potential to assist the clinicians during the entire epidural procedure, from the needle positioning to the catheter insertion monitoring inside the ES.

## 2. Materials and Methods

### 2.1. Sensorized Epidural Needle

**Figure 1:**
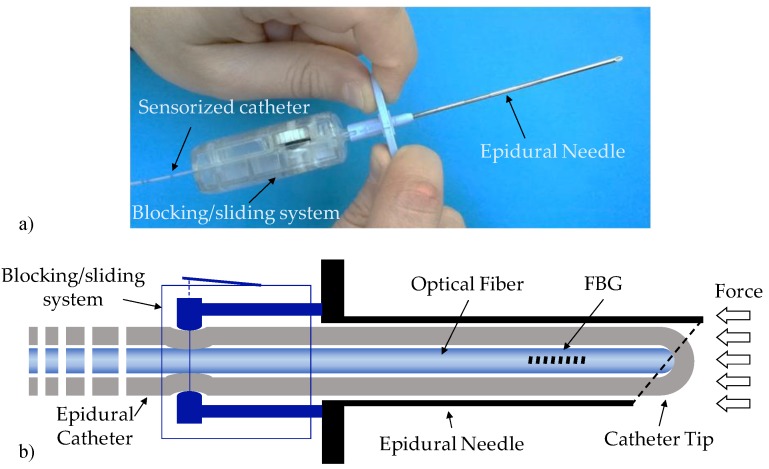
(**a**) Picture and (**b**) schematic of the developed device.

The developed guiding device is shown in Figure 1. The device basically comprises a standard 20 G nylon EC integrated inside the lumen of an epidural 18 G Tuohy needle and bound to it with a customized blocking/sliding system (BSS). The catheter is sensorized by means of an FBG (see Section 2.2), which measures the force exerted on the tip during the tissues penetration. The FBG is inserted inside the catheter lumen and locked in position through the BSS when it comes into contact with the closed tip of the EC (see Figure 1b). In fact, the distal tip of the EC is closed (lateral holes are used for drug administration) as open-end catheters are more likely to become blocked by blood clots during the insertion. The FBG is positioned at ~15 mm from the fiber end to maximize the probe sensitivity and avoid the fiber bending due to the curved shape of the Tuohy needle tip [11].

The BSS is a customized syringe-like device, which allows for both the locking and the sliding of the EC. It is very lightweight (~20 g) and it is connected to the epidural needle via a male luer lock connector. The BSS encloses a mechanical system mainly composed of a scroll wheel and a precision guide for the catheter. Once integrated, the sensorized EC can slide forward and backward by simply rotating the scroll wheel; therefore, it can be also easily taken off the needle without blocking up the lumen. At the initial position, the sensorized EC tip slightly protrudes from the needle tip in such a way that it comes directly in contact with biological tissues during penetration.

### 2.2. The Fiber Bragg Grating 

The FBG is a wavelength-selective optical filter with a very narrow bandwidth centered at a specific wavelength called the “Bragg wavelength” (λ_B_) [15]. As λ_B_ is a function of the grating period, any external perturbations (such as mechanical strain) acting on the fiber induce a Bragg wavelength shift. In this work, we used a single-ended FBG inscribed in a smf28e optical fiber coated with 15 µm thick polyimide layer. The FBG is 5 mm long and it is characterized by a Bragg wavelength of 1569.9 nm, a bandwidth of ~400 pm and a peak reflectance of ~−9 dB.

### 2.3. Console

The real-time monitoring of the Bragg wavelength is accomplished by means of a Micron Optics sm130 interrogator. This device employs full spectral scanning in the range of wavelengths from 1510–1590 nm with a sampling frequency of 1 KHz and a dynamic range of 25 dB. The interrogator is connected to a PC where the data (Bragg wavelength and peak intensity as a function of time) are displayed and recorded through a customized Labview^®^ plug-in. 

### 2.4. Calibration Tests

**Figure 2:**
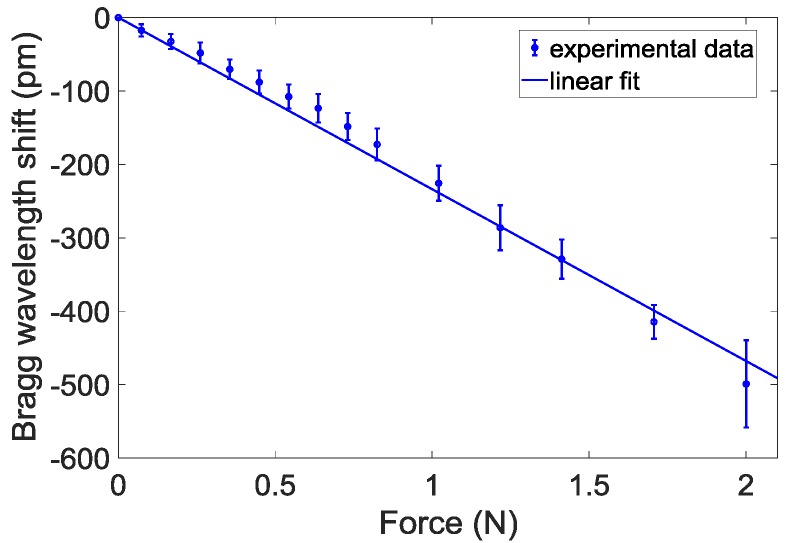
Calibration curve: wavelength shifts as a function of the weight force applied on the catheter tip.

We have carried out calibration tests to correlate the force variations applied on the catheter tip and the Bragg wavelength shifts. Details on both the calibration set-up and the procedure have been reported in our previous work [12]. Figure 2 shows the calibration curve obtained by applying a weight force in the range 0–2 N on the catheter tip. Results show an almost linear trend (as demonstrated by a linear correlation coefficient higher than 0.994) with a slope equal to −234 pm/N. The Bragg wavelength shift recorded at 2 N is ~500 pm. 

With a view towards catheter kinking detection, we also carried out experimental tests to estimate the minimum bending radius beyond which a significant intensity decrease of the Bragg peak occurs. The expected reflected peak drop is due to the intrinsic property of an optical fiber of inhibiting the light guidance if total reflectance condition is not satisfied [14]. Specifically, the sensorized EC was curved with curvature radius of 2.5, 5, 7.5 and 10 mm (with a degree of curvature of 90°) that induced Bragg peak reflection losses of −10.33 ± 0.38 dB, −3.4 ± 0.05 dB, −0.27 ± 0.02 dB and −0.11 ± 0.031 dB, respectively. These values are reported as mean ± standard deviation calculated over five observations. 

### 2.5. Experimental Methodology

In vivo tests were performed on a female porcine model of 7 months of age and 70 kg weight. The pig was positioned in the left lateral decubitus position on a fluoroscopy table and anesthetized with an intravenous injection of propofol 2 mg/kg, vecuronium 0.1 mg/kg, fentanyl 0.03 mg/kg and maintained with propofol infusion 4 mg/Kg/h, Ketamine 0.5 mg/kg/h. The pig was intubated and mechanically ventilated. Continuous monitoring of heart rate, pulse oximetry and blood pressure were performed throughout the experiment. At the end of the procedure, the pig was euthanized. The study was authorized by Italian Ministry of Health (auth. N. 554/2017) and was conducted at Cardarelli Hospital in Naples (Italy).

A skilled clinician performed three punctures in L5/L6, L6/L7 and L7/S1 spaces, following a midline approach. Similar to what was established in our previous work [12], the procedure started by inserting the needle only a few centimeters under the skin, until it was blocked inside the connective tissue. As the EC slightly protrudes from the needle tip, the FBG undergoes a compression once the needle is inserted inside the pig’s back. Then, the clinician did not apply any pushing force and waited about 10 s until the Bragg wavelength thermally stabilized at a specific value, which was set as the baseline [12]. The baseline is set at the beginning of each procedure, so that the force measurement during the needle penetration is self-referenced.

Once the baseline was fixed, the needle was advanced towards the ES, and the EC deformations occurring during the tissues penetration were recorded by monitoring the optical signal reflected from the FBG. The FBG thus ‘follows’ the catheter deformations, thus inducing Bragg wavelength shifts proportional to the force applied on its tip. The needle advancement was stopped when an abrupt force drop occurred and the signal value went below the baseline. The occurrence of both these events means that the needle is correctly placed inside the ES, as successively confirmed by X-ray scans.

Once the needle tip was correctly placed into the ES, the sensorized catheter was advanced by rotating the scroll wheel of the BSS, and the FBG reflectance peak was recorded in real-time. The catheter pulling and withdrawing was repeated several times, until the Bragg peak reflectance remained constant during the EC insertion. The absence of reflectivity losses means that the catheter is correctly placed (not kinked) inside the epidural canal, as confirmed by means of fluoroscopy analysis.

## 3. Results

### 3.1. Epidural Space Identification

**Figure 3:**
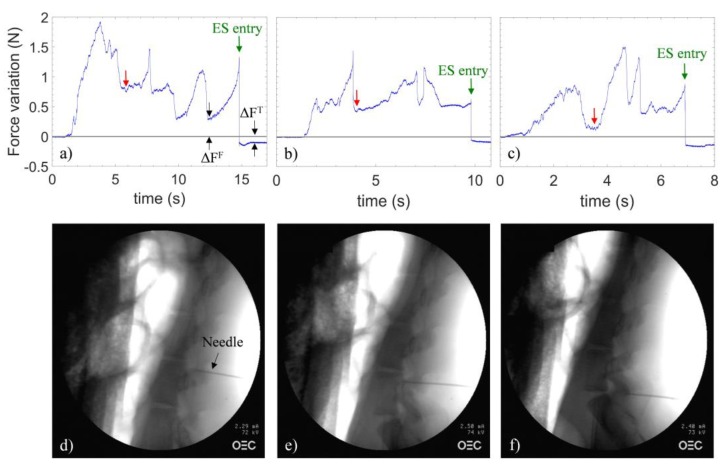
Signals recorded during in vivo testing in (**a**) L5/L6, (**b**) L6/L7 and (**c**) L7/S1 spaces. The gray line is the baseline; discrimination parameters (ΔF^T^ and ΔF^F^) are shown. X-ray images captured at the instant indicated by the green arrows in the figures pertaining to punctures in the spaces (**d**) L5/L6, (**e**) L6/L7 and (**f**) L7/S1.

Figure 3a–c shows the variations (with respect to the baseline) of the force applied on the catheter tip measured by the sensor during needle penetration in the spaces L5/L6, L6/L7 and L7/S1.

At time t = 0, the needle is inserted in the connective tissue. From this point forward, the clinician applies a continuous pressure to the needle. As the needle is advanced, the force variations depend upon the penetrated tissues rigidity. Specifically, a compression/relaxation of the EC occurs concomitant with a biological tissues stiffness increases/decreases, which, in turn, involves a blue/red shift of the Bragg wavelength, corresponding to positive/negative changes of the force with respect to the baseline.

It is not straightforward to match the single curve feature with each tissue boundary penetrated by the needle. This is because of the randomness of both the tissue consistency and the pushing force which is manually applied by the clinician on the needle. In any case, distinguishing all the tissue boundaries goes far beyond the scope of our work, which is carried out for the sole purpose of identifying the ES. Some common characteristics of the three curves can be identified. In fact, each force curve can be mainly divided into two parts. The first one starts with a positive force variation, which successively reduces after about 6, 4, and 4 s for the trace (a), (b) and (c) respectively (as pointed out by red arrows). Such a relaxation indicates that the needle tip is positioned in the space immediately preceding the ligamentum flavum. After this point, a second positive force variation occurs, vanishing with successive force drops, which sets the signal below the reference line (as indicated by green arrows). The presence of multiple force drops can be associated to the crossing of the two flavum layers [16]. Regardless of the number, the intensity, and the slope of the above-mentioned force drops, for the first time in the tests, a negative variation of the force occurs. Such a feature is effectively consistent with the ES entry [12]. Then, the clinician instantaneously stopped the procedure and the correct ES placement for each puncture was confirmed by X-ray scans, as shown in Figure 3d–f.

#### Discrimination Algorithm

**Figure 4:**
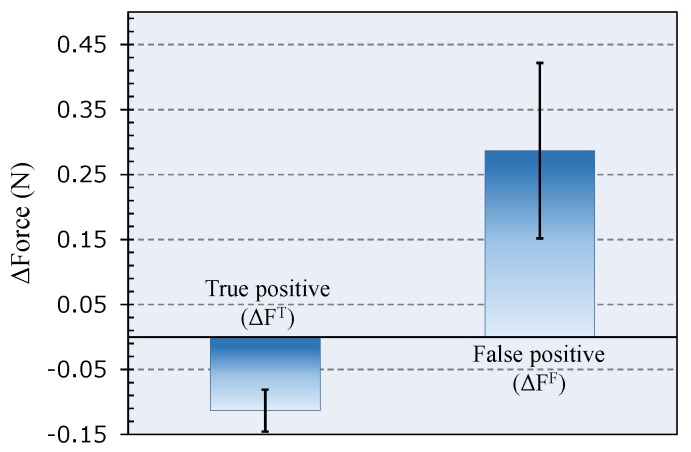
Force variation calculated as mean ± SD after a false (ΔF^F^) and true (ΔF^T^) positive event (details in the text); negative force variations have been recorded only when the needle is correctly positioned in the ES.

The repeated force drops might be wrongly interpreted as the entry into ES. However, in our measurements, the force variation (with respect to the baseline) registered when the needle enters the ES is unique and distinguishable because it is negative. This allows us to discriminate false from positive events. With reference to Figure 3, we indicated as ΔF^F^ (false positive) and ΔF^T^ (true positive) the minimum force variation (with respect to the baseline) registered immediately after an abrupt relaxation of the catheter, when the needle is outside and inside the ES, respectively. We evaluated ΔF^T^ and ΔF^F^ over the three tests; the results are shown in the histogram of Figure 4 as mean ± SD.

**Figure 5:**
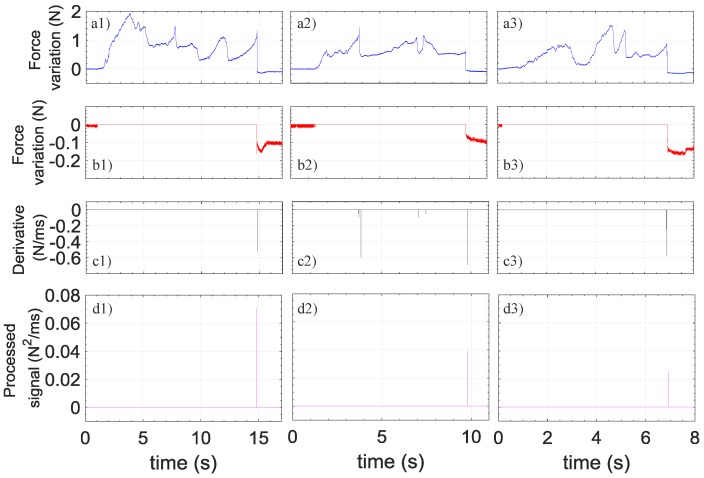
Force variations as a function of time recorded during penetration in L5/L6 (**a1**), L6/L7 (**a2**) and L7/S1 (**a3**) spaces; (**b1**) is the same as (**a1**) where positive values are set to zero; (**b2**) is the same as (**a2**) where positive values are set to zero; (**b3**) the same as (**a3**) where positive values are set to zero; (**c1**) is the derivative of (**a1**) where positive values are set to zero; (**c2**) is the derivative of (**a2**) where positive values are set to zero; (**c3**) is the derivative of (**a3**) where positive values are set to zero; (**d1**) is the processed signal given by the product between (b1) and (**c1**). (**d2**) is the processed signal given by the product between (**b2**) and (**c2**). (**d3**) is the processed signal given by the product between (**b3**) and (**c3**).

In order to easily and objectively distinguish true from false readings, a discrimination algorithm analyzing both the speed and the amplitude of signal variations has been implemented. The algorithm steps are shown in Figure 5. Briefly, the algorithm sets the positive values of the force variations (red curves) and of its derivative (black curve) to zero and then multiplies the signals obtained in the two previous steps, thus generating the final processed signals (magenta curves). The algorithm is able to provide a unique distinctive peak when the needle reaches the ES in all three cases. This result unequivocally demonstrates the ability of our probe to discern false from true positives.

### 3.2. Catheter Kinking Detection

**Figure 6:**
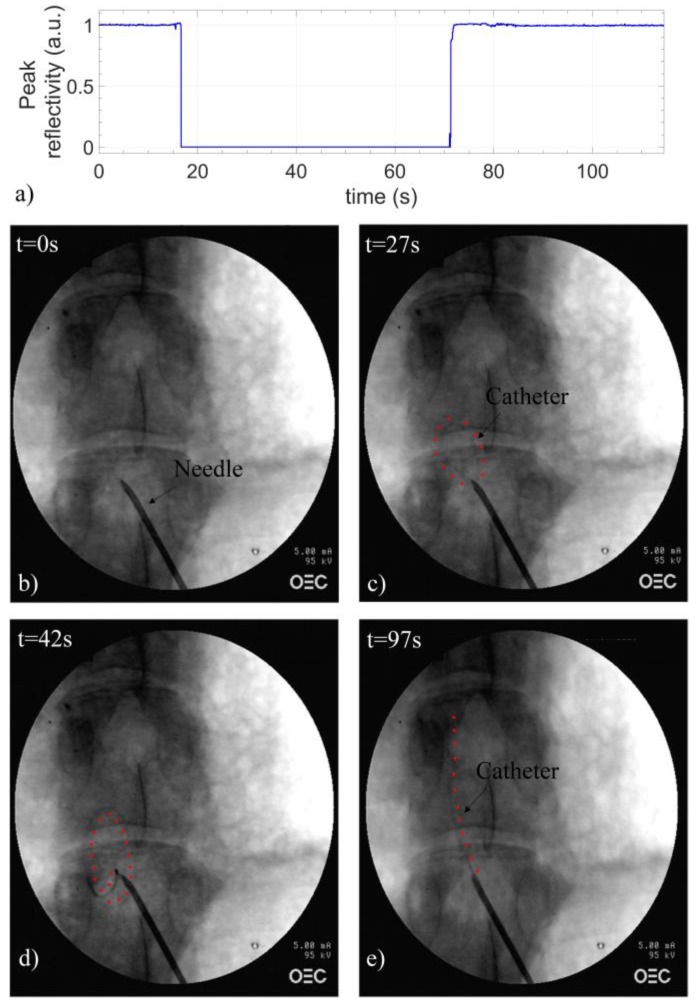
(**a**) Peak reflectivity as a function of time recorded during the insertion of the sensorized EC in L5/L6 space; X-ray images of the catheter (**b**) in the epidural needle and in different positions inside the ES: (**c**) curved, (**d**) coiled and (**e**) straight. Red dots, placed at the catheter’s side, highlight its trajectory.

Finally, we also demonstrated the potential of our device for detecting catheter kinking events. To this end, once the needle tip was correctly placed, the catheter was inserted inside the epidural canal while monitoring the maximum reflectance value provided by the FBG. By following the EC trajectory by means of X-ray fluoroscopy, we have found that, when the catheter undergoes kinking and coiling events, significant Bragg peak intensity drops are registered, which make the FBG response no more detectable by the interrogator used (SM-130). On the other hand, when the catheter follows its recommended trajectory and goes straight along the epidural canal, no significant reflection intensity drops occur. These results are resumed in Figure 6, where we show the Bragg peak intensity evolution and relatives X-ray images collected during the positioning of the EC inside L5/L6 space. During the first ~11 s, the FBG is still inside the needle lumen (Figure 6b); therefore, the reflectance value is equal to 1 a.u. As the EC is advanced, an abrupt signal drop occurred at ~17 s induced by the fiber bending; indeed, as demonstrated by the X-ray image shown in Figure 6c, the catheter is actually kinked and describes a sort of ellipse inside the ES. By restarting the advancement at 35 s, the fiber kept curving (Figure 6c) and no further signal increase was observed. For this reason, the probe was pulled off until a complete signal restoration occurred at ~72 s. Subsequently, the needle was rotated to prevent the catheter from hitting the epidural canal wall and the EC was advanced again. Different to the previous insertion, in this case, no signal loss was detected, thus entailing that the FBG did not bend. In fact, as confirmed by fluoroscopy (Figure 6e), the catheter is correctly positioned along the epidural canal.

## 4. Discussion

The results reported in the previous section demonstrate that the proposed device is able to effectively recognize the correct positioning of both the needle and the catheter inside the ES. Concerning the epidural needle placement, when the needle tip enters the ES, the sensorized EC suddenly relaxes and an abrupt drop of force and negative variation are perceived. This event does not occur in any of the other tissue boundaries previously penetrated by the needle, for which force variations are always positive. We developed a discrimination algorithm which provides a spike only when the needle enters the ES. When this distinctive sign occurs, a flashing light or an acoustic signal could alert the operator to stop the needle advancement.

The main benefits of using the proposed device for ES localization and the related technique have been already discussed in our previous works [11,12]; they include ease of use, conservation of medical technical practice and manual skill, and the possibility for the operator to use both hands without needing cumbersome instrumentation. With respect to the previous version of our device, in which the Hytrel-coated FBG was directly inserted inside the needle lumen, this novel configuration allows us to add further significant advantages. First, biocompatibility issues are fixed since the presence of the catheter avoids any physical contact between the optical fiber probe and the human tissues. Second, since the FBG is completely embedded inside the catheter, any accidental release of glass fragments in the case of breaks or damage inside the body is avoided.

Moreover, the same device can be also used for detecting incorrect catheter placement, thus preventing kinking or coiling events. In fact, we have found that, as the EC is advanced in the ES, the FBG peak value remains constant only when the catheter is correctly positioned (i.e., not curved) along the epidural canal; as a matter of fact, the coiling of the EC involves an abrupt signal decrease, which prevents the interrogator from detecting the FBG spectrum. It is important to remark that the catheter placement is currently a ‘blind’ procedure, especially for obstetrics for which the fluoroscopy cannot be used. Incorrect catheter placement seriously limits the success of epidurals and makes the entire clinical procedure ineffective. Our device allows us to overcome such a limitation. In fact, once that the catheter is correctly placed, by simply pulling off the fiber from the catheter lumen, it is possible to administer the anesthetic drugs through the catheter itself, as required by the current clinical procedure. Future catheter realizations could incorporate the optical fiber in the catheter walls in order to allow injection drugs without the need for removing the fiber probe [17]. Although the above discussed results are still preliminary, and further experimental tests are needed for improving the statistical significance of our observations, this study sets the stage for a new complete approach for epidurals, that guarantees the ES placement of both the needle and the catheter.

## 5. Conclusions

In this work, we have reported on a smart EC that is sensorized by integrating an FBG in its lumen. The catheter, and thus the FBG, suitably inserted inside the epidural needle, acts as a strain sensor able to continuously monitor the force variations applied on the needle tip during its penetration. The device has been first calibrated in lab and then tested in vivo on a porcine model. Results showed that our probe is able to ‘sense’ when the needle tip enters inside the ES: on overcoming the ligament flavum/ES boundary, an abrupt force drop is registered, which constrains the signal below a reference value (i.e., zero-force measured). According to our previous observations, such a feature represents a clear ES identification since it is unique and distinguishable from all the other force drops registered during penetrations (false positives). With respect to our first configuration that was based on the integration of a Hytrel-coated FBG inside the epidural needle lumen, this new version increases the level of patient safety due to the full biocompatibility and also improves the flexibility, since the catheter is already in position to be inserted inside the epidural channel to inject drugs. In fact, the same device allows us to prevent catheter kinking or coiling events which lead to ineffective analgesia. Indeed, we have demonstrated that the bending of the catheter inside the ES involves a strong decreasing of the FBG peak intensity. Therefore, by monitoring the intensity of the reflected signal, it is possible to retrieve an important feedback about the trajectory followed by the catheter during its advancement inside the epidural canal. Overall, the proposed device aims to become a complete guidance system to assist anesthesiologists during the entire clinical procedure, from the accurate localization of the ES to the correct placement of the EC.
